# Telomere-associated proteins add deoxynucleotides to terminal proteins during replication of the telomeres of linear chromosomes and plasmids in *Streptomyces*

**DOI:** 10.1093/nar/gkv302

**Published:** 2015-04-16

**Authors:** Chien-Chin Yang, Shu-Min Tseng, Carton W. Chen

**Affiliations:** 1 Department of Chemistry, Chung-Yuan Christian University, Chung-li 32023, Taiwan; 2 Department of Life Sciences and Institute of Genome Sciences, National Yang-Ming University, Shih-Pai, Taipei 11221, Taiwan

## Abstract

Typical telomeres of linear chromosomes and plasmids of soil bacteria *Streptomyces* consist of tightly packed palindromic sequences with a terminal protein (‘TP’) covalently attached to the 5′ end of the DNA. Replication of these linear replicons is initiated internally and proceeds bidirectionally toward the telomeres, which leaves single-strand overhangs at the 3′ ends. These overhangs are filled by DNA synthesis using the TPs as the primers (‘end patching’). The gene encoding for typical TP, *tpg*, forms an operon with *tap*, encoding an essential telomere-associated protein, which binds TP and the secondary structures formed by the 3′ overhangs. Previously one of the two translesion synthesis DNA polymerases, DinB1 or DinB2, was proposed to catalyze the protein-primed synthesis. However, using an *in vitro* end-patching system, we discovered that Tpg and Tap alone could carry out the protein-primed synthesis to a length of 13 nt. Similarly, an ‘atypical’ terminal protein, Tpc, and its cognate telomere-associated protein, Tac, of SCP1 plasmid, were sufficient to achieve protein-primed synthesis in the absence of additional polymerase. These results indicate that these two telomere-associated proteins possess polymerase activities alone or in complex with the cognate TPs.

## INTRODUCTION

**Figure 1.**
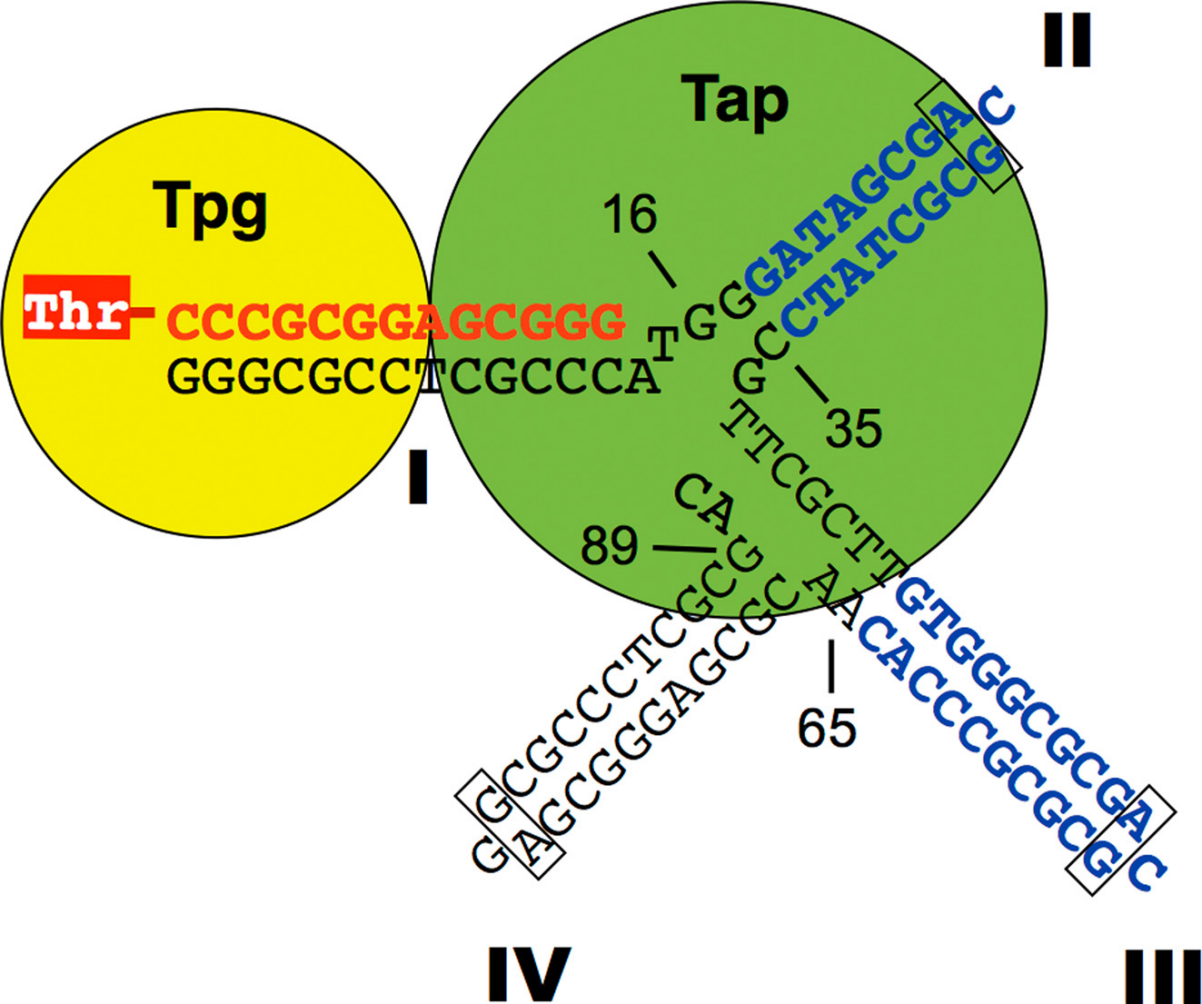
The 3′ overhang of the typical telomeres of *Streptomyces*. Shown are the first 91 nt that span the first four palindromes (Roman numerals). The last nt (16, 35, 65 and 89) of four templates used in this study are indicated. Palindromes II to IV are folded into hairpins. The G:A sheared pairings are boxed. The Tap binding sequences in Palindromes II and III are marked in blue. Palindrome I is duplexed with the 13-nt oligonucleotide primer (in red) synthesized from the Tpg primer (yellow disk). For an approximate mechanistic perspective of the molecular interactions, the two proteins are assumed to be globular and drawn to scale based on the diameters (Tpg: 39.3 Å; or 11.5 bp; Tap: 62.4 Å or 18.4 bp) estimated from their amino acid contents. Tap is placed over its binding sequence at Palindrome II, and bound to Tpg (9), which, in turn, is covalently linked to the first nucleotide C of the primer through a phosphodiester bond (dash line) at the Thr-114 (red shaded ‘T’).

Soil bacteria *Streptomyces* possess linear chromosomes and linear plasmids. Replication of these linear replicons starts from an internal origin, and proceeds bidirectionally toward the telomeres, leaving single-stranded overhangs at the 3′ ends that need to be patched reviewed in (1). The sequences of the 3′ single-stranded overhangs, about 300 nt in lengths (2), contain several tightly-packed palindromic sequences with potentials to form very stable secondary structures (3,4; Figure 1). For most of the *Streptomyces* chromosomes and some linear plasmids, these palindromic sequences are highly conserved (designated ‘typical telomeres’). In the typical telomeres, Palindrome I, consisting of the first 13 nt, is complementary to part of Palindrome IV. Between these two palindromes, Palindromes II and III each form a hairpin structure closed by stable G:A sheared pairing (boxed, Figure 1). These structures resemble the ‘rabbit ear’ structures seen at the 3′ end of autonomous parvovirus genomes (5), which are essential features for replication.

During replication, the single-stranded gaps at the *Streptomyces* telomeres are filled up by DNA synthesis primed by terminal protein (TP), which remains covalently bound to the 5′ end of DNA (reviewed in 1). The fill-in process is termed ‘end patching’. The TPs that cap the typical telomeres (‘typical TPs’) is encoded by *tpg*, which forms an operon with a gene encoding a telomere-associated protein (*tap*) in the terminal regions of most *Streptomyces*. Both *tpg* and *tap* are essential for replication of the typical telomeres of *Streptomyces*.

Among most *Streptomyces* species, Tpg is highly conserved in size (mostly 185 aa) and sequence with a very high pI. It contains a DNA-binding thumb domain of HIV reverse transcriptase (6,7) and a functional nuclear localization signal (8). Tap is also highly conserved, and contains a DNA binding helix-turn-helix domain. Other than that, there is no similarity to any known protein. The function of Tap is unknown, except that binds specifically to Palindromes II and III in the telomere sequence, and interacts strongly with Tpg to form a strong telomere complex (9). It has been suggested that the function of Tap is to bind the single-stranded overhang and tether Tpg to the correct position for DNA polymerase to act on (9).

A minority of linear *Streptomyces* replicons contains atypical telomere sequences. The first characterized atypical telomeres are those of SCP1 plasmid. The SCP1 telomeres start with a stretch of dGMP, and are capped by an atypical terminal protein (Tpc). The encoding gene, *tpc*, also form an operon with *tac*, whose product also binds Tpc (10). Both Tac and Tpc are essential for replication of SCP1.

TP-primed replication of phage ϕ29 employs a B-family DNA polymerase for catalysis. However, *Streptomyces* genomes do not encode any B-family DNA polymerase. Instead, they encode five other DNA polymerases—Pol I, DnaE1, DnaE2, DinB1 and DinB2. Of these enzymes, either one of the family Y (translesion synthesis) DNA polymerases, DinB1 or DinB2, is required for the end patching during replication (11). At least one of the two polymerases is required for the replication of linear, but not circular, replicons in *Streptomyces*.

We have previously established an *in vitro* deoxynucleotidylation system, in which Tpg was specifically labeled by radioactive dCTP, the first nucleotide of the chromosomal DNA (12). In this system, crude cell extract was used to provide the source of polymerase activity. Here we attempt to substitute the cell extract with purified DinB1. Surprisingly, we discovered that DinB1 was not necessary for the deoxynucleotidylation reaction. Instead, Tap was found to be required and sufficient for the deoxynucleotidylation of Tpg. Moreover, in the presence of all four dNTPs, the incorporation was extended to 13 nt, corresponding to Palindrome I of the typical telomere. All these indicate that end patching synthesis is initiated by synthesis of a short DNA primer from the protein primer (TP) by the concerted action of Tap and Tpg, which is then further extended by a DNA polymerase (presumably DinB1 or DinB2). This scenario is analogous to the initiation of replication of Hepatitis B virus by the viral-encoded P protein, of which a reverse transcriptase domain synthesize an DNA primer on its TP domain (13). In both cases, no RNA primer is involved.

## MATERIALS AND METHODS

### Protein expression, purification and analysis

*tap, tpg* and *dinB1* were tagged with His-6 at the N-terminus, cloned in pET15 vector (Stratagene) and expressed in *Escherichia coli* BL21(DE3). The His-tagged proteins were purified in a His Tag Binding Agarose column (Bioman, Taiwan). DinB1, which formed inclusion bodies, was purified in the presence of urea, which was then removed by dialysis (14). To improve the purity of these protein, the materials that bound to the column was washed in twice more volume of washing buffer containing up to 1 M NaCl. The proteins were then eluted in 20 mM Tris–HCl buffer (pH7.9) containing 10% glycerol, 1 M or 0.5 M NaCl and 500 mM imidazole. The concentrations of the purified proteins were determined by Bradford method against bovine serum albumin (BSA), and the proteins were stored in aliquot at −20°C.

Tap was further purified by binding to the 35-nt polynucleotide of the 3′ end of the typical telomere (containing Palindromes I and II; Figure 1) that is linked to biotin at the 3′ end with a 10-nt spacer. The Tap protein eluted from the His Tag Binding Agarose column was mixed in 150 μM polynucleotide-biotin, and 0.5 mg/ml sheared salmon sperm DNA in K buffer (10 mM Tris–HCl, pH 7.5, 7 mM Mg^2+^, 0.1 mM dithiothreitol), and incubated for 30 m at 25°C. The mixture was then mixed with K buffer containing streptavidin magnetic particles (SA-PMP, Promega) and incubated for another 1 h with gentle rotation. After washing, Tap was eluted in A8 buffer (50 mM Tris–HCl, pH 8.0, 10% glycerol) containing various concentration of NaCl. Most Tap was eluted at 500 mM NaCl, which was checked for purify with electrophoresis and silver staining, and used for end patching reactions.

### *In vitro* priming system

Double-stranded DNA templates were generated by PCR (see Supplementary Material Table S1) or cleaved from clones in a TA-vector. Single-stranded templates (telomeric overhang, TO) were generated by heating the double-stranded templates at 98°C for 20 min or synthesized directly. Typical priming reaction mixtures (in 20 μl) contained single-stranded or double-stranded templates (in various concentrations), 60 nM Tap, 600 nM Tpg, 0.165 μM [α-^32^P]-dCTP (2 μCi), 1.0–1.5% glycerol, 50–75 mM NaCl and 50–75 mM imidazole in K buffer. For extension of oligonucleotides, non-radioactive dNTPs prepared in deionized water were added. The reaction mixture was typically incubated at 30°C for 10 min, and stopped by filtration through a G-25 spin columns pre-equilibrated with 0.1% sodium dodecyl sulphate (SDS) (for subsequent electrophoresis) or de-ionized water (for further manipulations). Reaction products were analyzed on 12 or 15% sodium dodecyl sulphate-polyacrylamide gel electrophoresis (SDS-PAGE) directly or after heated in 90°C hot water 15 m. After electrophoresis, the gel was dried and subjected to autoradiography (15). Radioactivity was determined in Phosphoimager or Cerekov count of sliced dried gel.

Treatments of the reaction products with Exo III or T7 endonuclease I (NEB) and other restriction enzymes were carried out according to the specifications provided by the manufacturers. Proteinase K (Sigma) treatment was carried out in 20 mM Tris–HCl buffer (pH 8.5) containing 1 mM Ca^2+^ and 1% SDS at 37°C for 1 h. TP was separated from the telomere DNA by incubation in 1 N NaOH 37°C for 2 h.

## RESULTS

### Deoxynucleotidylation of TP is achieved *in vitro* in the absence of added DNA polymerase

In our previous *in vitro* deoxynucleotidylation system, the *Streptomyces* cell extract was used as the source of polymerase activity. Since the two translesion DNA polymerases, DinB1 and DinB2 have been shown to be important for end patching of the *Streptomyces* telomeres (11), we set out to establish an *in vitro* system using purified DinB1 in place of the cell extract. For the reaction, the single-stranded telomeric overhang (abbreviation ‘TO’) of the *Streptomyces lividans* chromosome of various lengths was used as the template.

**Figure 2.**
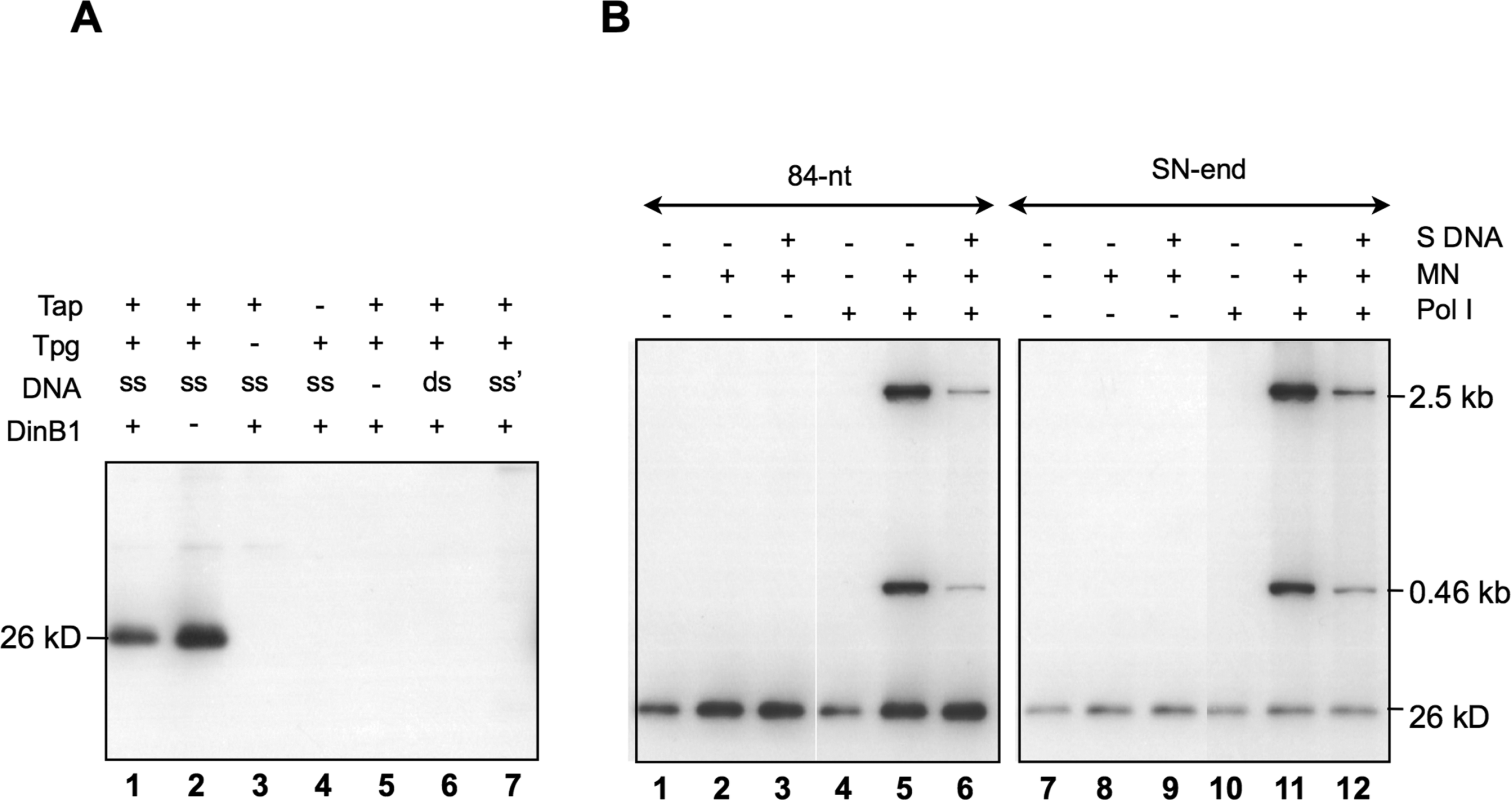
*In vitro* deoxynucleotidylation and oligonucleotide elongation. (**A**) *Deoxynucleotidylation in the presence or absence of DinB1*. The reaction mixture contained 0.165 μM [α-^32^P]-dCTP, 1.5% glycerol, 75 mM NaCl, 75 mM imidazole, and various concentrations of proteins in K buffer (10 mM Tris–HCl pH 7.5, 7 mM Mg^2+^, 0.1 mM DTT). The final concentrations of proteins used were 135 nM of Tap, 280 nM of Tpg and 500 nM of DinB1. The template was a 200-bp PCR-generated *S. lividans* telomere DNA digested by HaeIII digestion, which left the very end of the telomere sequence exposed (‘ds’), or its heat denatured form (‘ss’) in a final concentration of 9 nM. The products were separated by 12% SDS-PAGE. The radioactively labeled deoxynucleotidylated Tpg exhibited a mobility of a 26 kD protein. In lane 7, the fragment (‘ss’) was the 200-bp PCR product without HaeIII digestion. (**B**) *Deoxynucleotidylation and oligonucleotide extension in the absence of DinB1*. The reaction mixture contained 0.165 μM [α-^32^P]-dCTP, 1% glycerol, 50 mM NaCl, 50 mM imidazole, 60 nM Tap, and 600 nM Tpg (and no DinB1). In some cases, the following materials were added: two MulI–NdeI restriction fragments (‘MN’; 2.5- and 0.46-kb), salmon sperm DNA (‘S DNA’, 2 μg) and *E. coli* Pol I (10^−2^ units). The template was an 84-nt telomere DNA (50 nM) or a 460-bp SalI–NdeI (‘SN-end’) fragment containing the telomere DNA of *S. lividans* with the end exposed at the SalI cleavage site at a concentration of 1.35 nM.

DinB1 with a N-terminal His-tag was expressed in *E. coli*, and purified. The purified DinB1 was added to an *in vitro* deoxynucleotidylation reaction mixture containing Tpg (the substrate), Tap, a 200-nt single-strand TO (designated TO200), and [α-^32^P]-dCTP. The reaction yielded, after Exo III treatment, a radioactive product of an apparent molecular weight of 26 kD (Figure 2A, lane 1), which presumably represented deoxynucleotidylated Tpg (22 kD).

The deoxynucleotidylated product was not seen in the controls, in which either Tpg (lane 3), Tap (lane 4), or the template DNA (lane 5) was omitted. No deoxynucleotidylation was observed also, if the template DNA was in duplex (lane 6). This was in agreement with the previous observation that Tap binds preferentially to the hairpin structures formed by the denatured telomere DNA (9). Moreover, no product was observed, if the very end of the telomere sequence was not exposed (by HaeIII cut; lane 7).

Unexpectedly, when DinB1 was omitted in the reaction, the same deoxynucleotidylated product appeared (lane 2). It was expected that the DNA polymerase would be required for the deoxynucleotidylation reaction. There existed a possibility that, despite the extensive purification steps, the purified Tpg, Tap or DinB1 preparations might be contaminated by an *E. coli* DNA polymerase, most likely the most abundant DNA polymerase I (Pol I, accounting for >95% of the polymerase activity). No protein of the molecular weight of Pol I (109 kD) could be detected in these preparations by SDS-PAGE and coomassie blue staining or silver staining, which detected 10^−2^ U of Pol I in our system (data not shown).

To test the possible involvement of an *E. coli* DNA polymerase further, we added the *E. coli* Pol I to the reaction. The results (Figure 2B, lanes 1, 4) showed no increase in Tpg deoxynucleotidylation. Two restriction fragments (2.5- and 0.46-kb MluI and NdeI fragments of plasmid MS) were added to the reaction. The 5′ protruding ends of the MluI cuts provided a substrate for fill-in reaction by Pol I. These fragments were not labeled in the reaction mixture in the absence of added Pol I; neither did they compete with deoxynucleotidylation of Tpg (lane 2). The addition of salmon sperm DNA also did not affect the deoxynucleotidylation (‘S DNA’; lane 3). These results indicated the absence of detectable *E. coli* DNA polymerase activities in our preparations of the *Streptomyces* proteins.

To rule out the possibility that the telomere DNA in the reaction might compete for Pol I and thus reduced the labeling of the restriction fragments, the concentration of the denatured telomere DNA was reduced by ca. 40 folds (lanes 9–12) in the reaction. The results still showed a lack of labeling of the restriction fragments.

In comparison, when Pol I was also added, the 2.5- and 0.46-kb MluI and NdeI fragments were labeled as expected (lane 5). Addition of excess of salmon sperm DNA as a competitor significantly diminished the radioactivity labeling of the two restriction fragments but not deoxynucleotidylation of Tpg (lane 6).

### Tap adds a short sequence of DNA to Tpg

**Figure 3.**
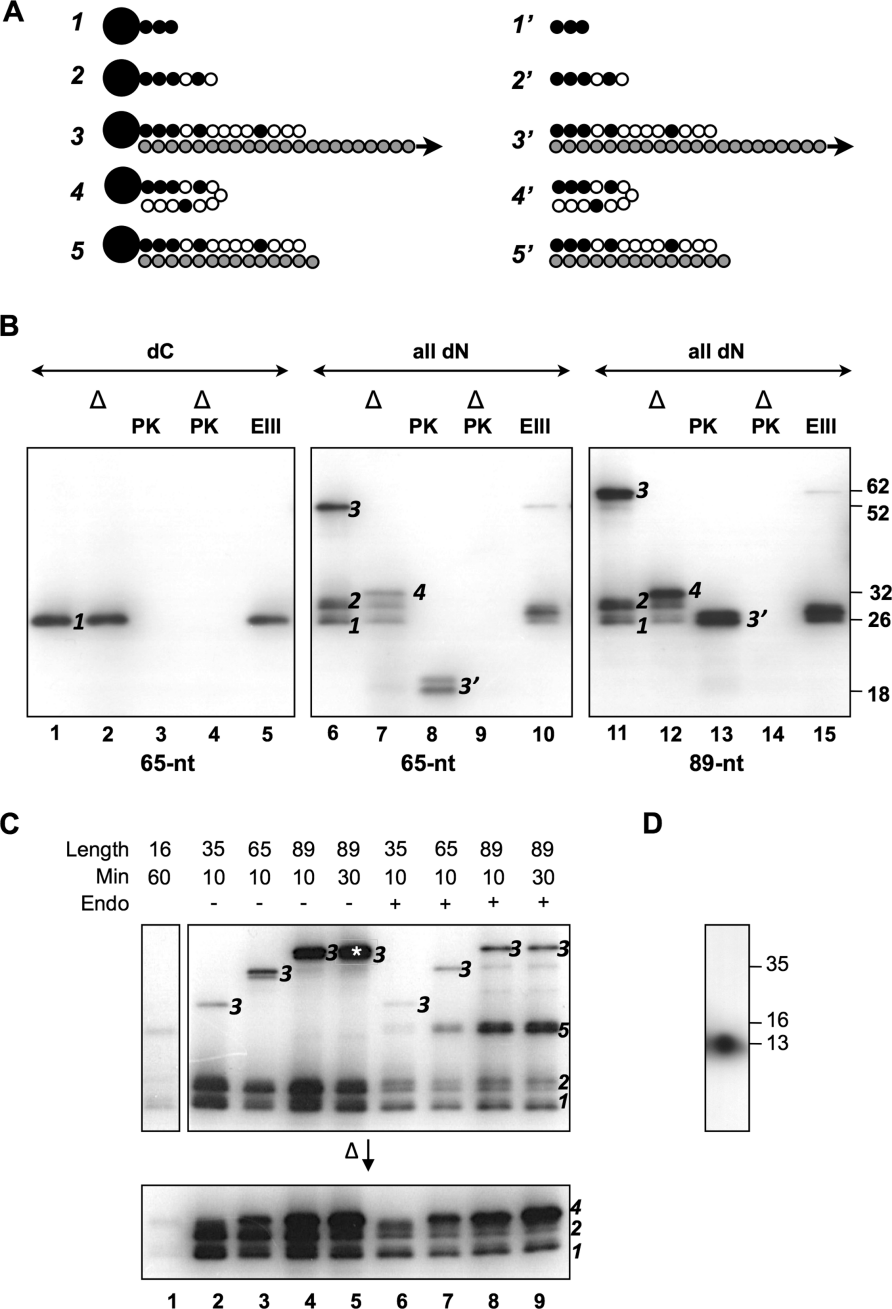
Characterization of the elongation products produced. (**A**) *Different forms of products of oligonucleotide elongation*. Form *1*. Tpg (large filled circle) linked to 1–3 radioactive dCMP (small filled circle). Form *2*. Tpg linked to radioactive dCMP and non-radioactive dNMP (open filled circle) with the total length not exceeding 7 nt. Form *3*. Tpg linked to13 nt-oligomer (Palindrome I) complexed with the template strand (shaded circles), the length of which is not to scale. Form *4*. Tpg attached to the 13-nt oligonucleotide (which fold back to form a hairpin) without the template, representing the denaturation product of Form *3*. Form *5*. Tpg linked to 13-nt oligomer duplexed with the template strand of 14–16 nt, representing T7 endonuclease I digestion product of Form *3*. Form *1*′–*5*′ proteinase K digestion products of Form *1*–*5*. Tpg was removed from the oligonucleotides. (**B**) *The products of the first step of end patching based on TO65*. The template was 65- (left and middle panels) or 89-nt (right panel) telomere DNA. The reaction conditions were as in Figure 2B except that the product was eluted from the G-25 spin column in deionized water. The eluted product was subjected to proteinase K (‘PK’) digestion, Exo III (‘EX’) digestion, or heat denaturation (‘Δ’). *Left panel*. Only radioactive dCTP (‘dC’) was included. *Middle panel*. Radioactive dCTP and non-radioactive dATP, dTTP and dGTP (‘all dN’) were included. The different forms of products identified in the SDS gels were marked. The sizes of marker proteins (in kD) are indicated to the right. (**C**) *The products produced on templates of different lengths. Upper* panel. The reactions were carried under the same conditions as described in (B) with all dNTPs added. The lengths of the template strands used are as indicated. In three cases, the incubation time was increased from 10 m to 30 and 60 m as indicated. Some products were further treated with T7 endonuclease I (‘Endo’). *Lower panel*. The products were heat-denatured before being subjected to electrophoresis. The various forms of the products are marked in the gels. The product, which was eluted for further analysis in (D) is marked by an asterisk. (**D**) *Sizing the length of the oligonucleotide*. Form *3* produced from the 89 nt template (marked with an asterisk in (C) was eluted from the gel slice in 0.1% SDS by soaking. The eluent was concentrated by ethanol precipitation, re-dissolved in 1 N NaOH, and incubated at 37°C for 2 h. After neutralization with HCl, the sample was electrophoresed in a 15% urea–PAGE at 20 V/cm together with three size markers—single-stranded DNA of TO13, TO16 and TO35 as indicated. The positions of the size markers were determined by UV absorption and EtBr staining. The position of the products was determined by autoradiography.

To see whether Tap might carry out DNA synthesis beyond the first three deoxycytidine residues, the other three dNTPs were added to the reaction, in which a synthesized 65-nt *S. lividans* telomere (‘TO65’) was the template. The reaction products were subjected to analyses by heat denaturation and proteinase K digestion. Various forms of products were expected with different added dNTPs (Figure 3A).

With only dCTP (‘dC’) added, the product of approximately 26 kD (Figure 3B, lane 1), designated Form *1*, was resistant to heat denaturation (lane 2), indicating that it was single-stranded. It disappeared upon proteinase K treatment (lanes 3 and 4; Form *1*′ in A), indicating that Form *1* represented Tpg linked to one of more dCMPs attached. On the other hand, Form *1* was resistant to ExoIII digestion (lane 5), indicating that the incorporated nucleotides were too short (perhaps 1–3 nt) to be susceptible to the enzyme digestion.

When all four dNTPs were added to the reaction, three products were observed (lane 6)—Form *1*, a nearby product of ∼30 kD (designated Form *2*), and a product migrating much more slowly (designated Form *3*). Upon heat denaturation (lane 7), Form *2* appeared to maintain the same mobility, indicating the absence of attachment to the template, whereas Form *3* exhibited a much higher mobility of ∼32 kD (Form *4*), most likely due to the disassociation from the template.

Thus, Forms *2* and *3* represented Tpg linked to newly synthesized oligonucleotides without and with the template attached, respectively. Thermodynamic calculation based on base stacking energy, and the specific concentrations of Mg^2+^ (7 mM), Na^+^ (75 mM) and template DNA (50 nM) shows that the template-nascent oligomer duplex would reach a *T*_m_ higher than 25°C only when the nascent oligomer is more than seven nt in length (Supplementary Materials Figure S1). Thus, Form *2* should represent Tpg linked to nascent oligomers shorter than 7 nt, and Form *3* should represent Tpg linked to the oligonucleotides longer than 7 nt and duplexed with the template. This would also account for the large gap between mobility of these two forms before denaturation (land 6) but a narrow difference in mobility after denaturation (lane 7).

Treatment of proteinase K (lane 8) would remove the bulk of Tpg from the labeled oligonucleotides. For Forms *1* and *2* the resulting labeled oligonucleotides of <7 nucleotides were too small to be detected in the electrophoresis run. The digested product of Form *3* would be the oligonucleotides perhaps attached to a small peptide and the template (Form *3*′) exhibited a higher mobility. When the sample was treated with both proteinase K and denaturation, Form *3* also disappeared together with the other forms as expected (lane 9). Finally, Exo III digestion reduced both Forms *2* and *3* to about the same size as Form *1* as expected (lane 10).

Similar results were obtained using a larger 89-nt telomeric overhang (TO89) of *S. lividans* (lane 11–15). These results indicated that Tap was able to carry on DNA synthesis beyond the first deoxycytidine.

### Tap fills in the first 13 nucleotides

The minimum length of the typical telomeres required for end replication was 167 bp, which spans the first seven palindromes (16), and Tap binds to the loops formed by Palindromes II and III (9). A series of templates of different sizes, from 16 to 89 nt (TO16, TO35, TO65 and TO89), was tested for the fill-in reaction by Tap (Figure 3C).

The 16-nt template, covering only the first palindrome, was able to support detectable radioactive incorporation, if the concentrations of the reactants and reaction time were significantly elevated (lane 1). This suggested that, in the absence of its cognate binding sequences (in Palindromes II and III), Tap was able to find the substrate and template for the reaction at a lower efficiency.

With the increases in template size, Forms *1* and *2* remained the same as expected from their lack of association with the template strand (lanes 2–4). On the other hand, the Form *3* products decreased in mobility with the increase of template size, and heat denaturation brought them to near the other two forms as expected from the removal of the template.

The level of nucleotides incorporation also increased with the increased template size. The 89-nt template that contained all the first four palindromes gave the best yield. Prolonged incubation resulted in increased incorporation, but not the size of the products (lane 5). This suggested the presence of a block for elongation at a specific site.

Digestion of the products with T7 endonuclease I, which recognizes and cleaves non-perfectly matched DNA, produced a new fragment in the reactions that contained TO35, TO65 and TO89 templates (lanes 6–9). The GCA loops in Palindromes II (and III) are closed by G-A sheared pairing, which is resistant to single-strand specific nucleases (4). We interpreted this product, designated Form *5* (Figure 3A), to be generated by cleavage of the labeled product at the junction between Palindromes I and II (Figure 1). This suggested that the oligonucleotide synthesis did not extend beyond the junction between Palindromes I and II, leaving it exposed to T7 endonuclease I.

To estimate the size of the oligonucleotide synthesized in this system, the Form *3* DNA from the reaction using TO89 template was extracted from the gel and subjected to alkaline treatment, which would remove Tpg and denature the DNA. The resulting single-stranded DNA was subjected to electrophoresis in a sequencing gel, and the results showed a molecule of approximately 13 nt long (Figure 3D), in agreement with the previous suggestion.

### The Tap-synthesized oligomer matches the telomere sequences

**Figure 4.**
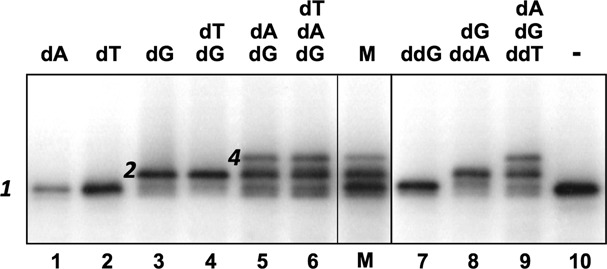
The dictation of the extending oligomer sequence by the template sequence. The reaction conditions were as in Figure 2B. The template was the 89-nt telomere DNA. Non-radioactive dNTP and ddNTP was added to a final concentration of 1 μM each. The products were heat denatured before electrophoresis. Lane M contains a mixture of products in lanes 1–3 as size markers.

To determine whether the synthesized oligomer was indeed copied from the template sequence, different combinations of deoxynucleotide triphosphates were added with radioactive dCTP to the reaction mixtures to test the effects on the extension (Figure 4). Adding dATP or dTTP did not affect the size of the product (lanes 1 and 2). On the other hand, addition of dGTP increased the size of the product (lane 3). This was expected from the telomere sequence, of which the first seven nucleotides are composed of only G and C (Figure 1).

Addition of dTTP together with dGTP did not alter the product (lane 4), but addition of dATP with dGTP further increased the size of the oligomer (lane 5). This was also expected from the telomere sequence. Interesting, adding dTTP to the latter did not cause any further change (lane 6), although T is the next nucleotide to incorporated according to the sequence. This again indicated that the oligonucleotide stopped after the first 13 nt (Palindrome I).

Dideoxynucleotides were also used for further tests. Addition of ddGTP did not affect the incorporation of radioactive dCTP (lane 7). Addition of ddATP did not affect the incorporation of dCTP and dGTP (lane 8). Addition of ddTTP did not affect the incorporation of the other three dNTP (lane 9). These are all consistent with the sequence of incorporation of these nucleotides based on the template (Figure 1).

### Manganese ion strongly promotes deoxynucleotidylation of Tpg

Mn^2+^ has been known to stimulate *in vitro* deoxynucleotidylation of TP primers in adenovirus (17), ϕ29 (18), PRD1(19) and hepadnaviruses (20), but not the subsequent elongation reactions (being inhibitory in some cases).

**Figure 5.**
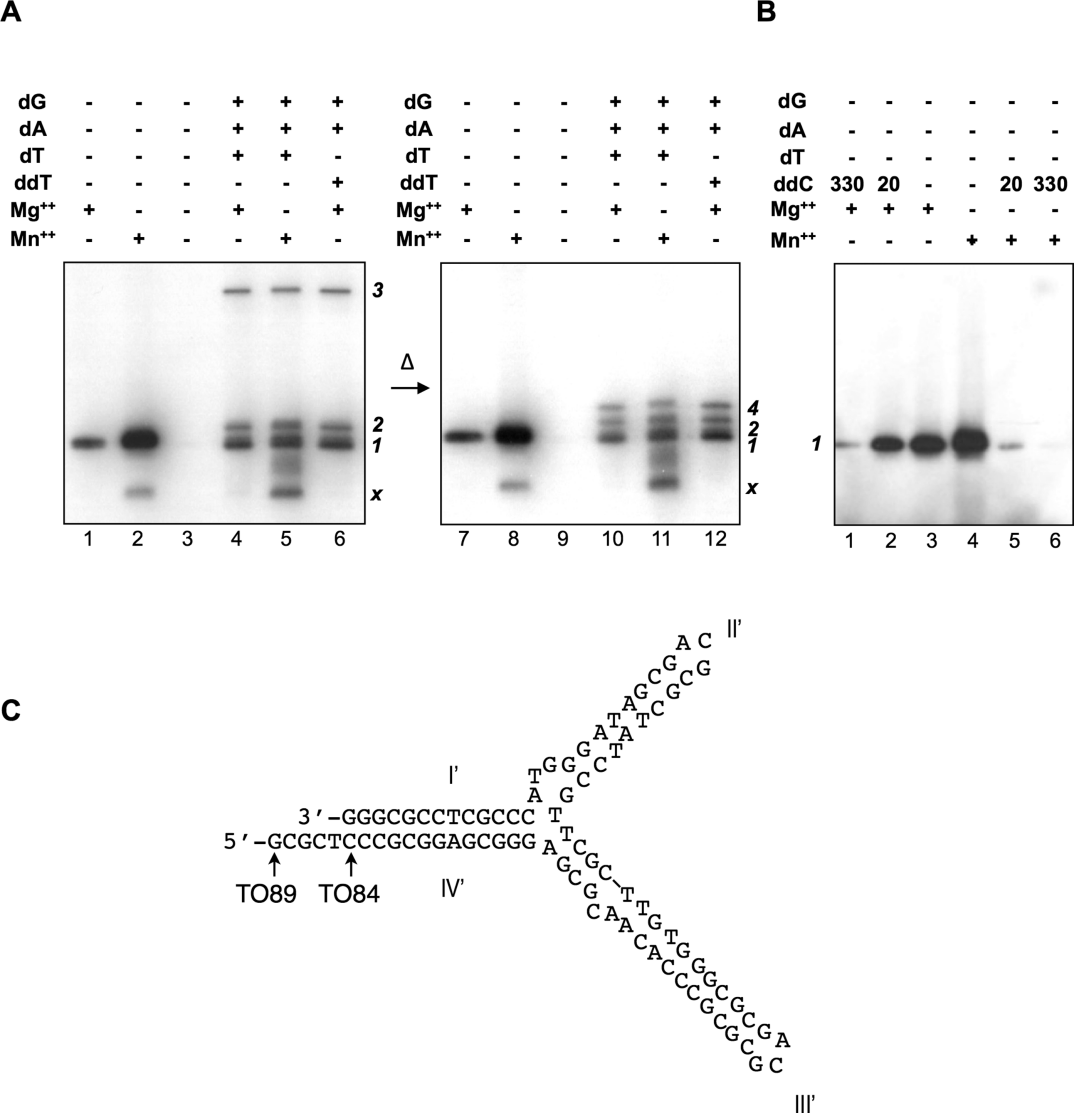
Effect of Mn^+2^ on deoxynucleotidylation and oligonucleotide elongation. (**A**) *The effect of Mn^+2^. Left panel*. The template used was the TO89. The reactions shown in lanes 1, 2 and 3, contained only [α^32^P]-dCTP precursor. The others were supplemented with the three non-radioactive dNTPs (1 μM each; lanes 4 and 5) or dGTP (‘dG’), dATP (‘dA’) and ddTTP (‘ddT’; lane 6). The reactions in lanes 2 and 5, Mg^2+^ (7 μM) was substituted with Mn^2+^ (1 μM), and that in lane 3 contained no divalent metal ions. *Right panel*. The same samples were heat denatured before electrophoresis. The different forms of products (*1, 2, 3, 4, x*) present in the gels are marked. (**B**) *The effect of ddCTP*. ddCTP (‘ddC’) was added in different (0, 20 and 330 n*M*) in the presence of Mg^+2^ (lanes 1–3) or Mn^2+^ (lanes 4–6). No non-radioactive dGTP, dATP or dTTP (‘dT’) was added. (**C**) The predicted secondary structure formed by TO89. The nt at the 5′ end of TO89 and TO84 are indicated by the arrows.

Similar results were obtained, when we substituted Mn^2+^ for Mg^2+^ in our reaction mixtures (Figure 5A). The deoxynucleotidylation of Tpg (Form *1*) was strongly stimulated (lane 2), while the elongation of the oligonucleotide (Form *3*) was not affected (lane 5). In addition, a new form (designated Form *x*) was produced in the presence of Mn^2+^. The electrophoretic mobility of Form *x* was higher than Form *1*, and was insensitive to heat denaturation (lanes *8* and *11*) or proteinase K (data not shown). The results indicated that Form *x* was a single-stranded oligonucleotide extended from a DNA primer. The only 3′ end available for extension was the 3′ end of Palindrome I on the template DNA. Thermodynamically, Palindrome I may fold back to Palindrome IV or to itself (less stably) to form stable secondary structures. Such fold-back structure may provide ground for extension synthesis from the 3′ end of Palindrome I. In the case of TO89, fold back of Palindrome I on Palindrome IV yielded a 5-nt 5′ protruding for fill-in (Figure 5C). To test whether Form *x* represented such a fill-in product, we performed the same reaction using a shorter template TO84 (Figure 5C), which lacked the 5′ protruding for fill-in. No Form *x* was detected in the product of this reaction (Figure 5B, lane 4) in support of our supposition.

Mn^2+^ has been shown to lower nucleotide discrimination by DNA polymerases against dideoxynucleotides (21). Whether Mn^2+^ may result in lower discrimination against ddCTP in deoxynucleotidylation by Tap was also tested. The results (Figure 5B) showed a strikingly low (perhaps no) discrimination against ddCTP.

### The 13-nt primer is extended by DNA polymerase I

**Figure 6.**
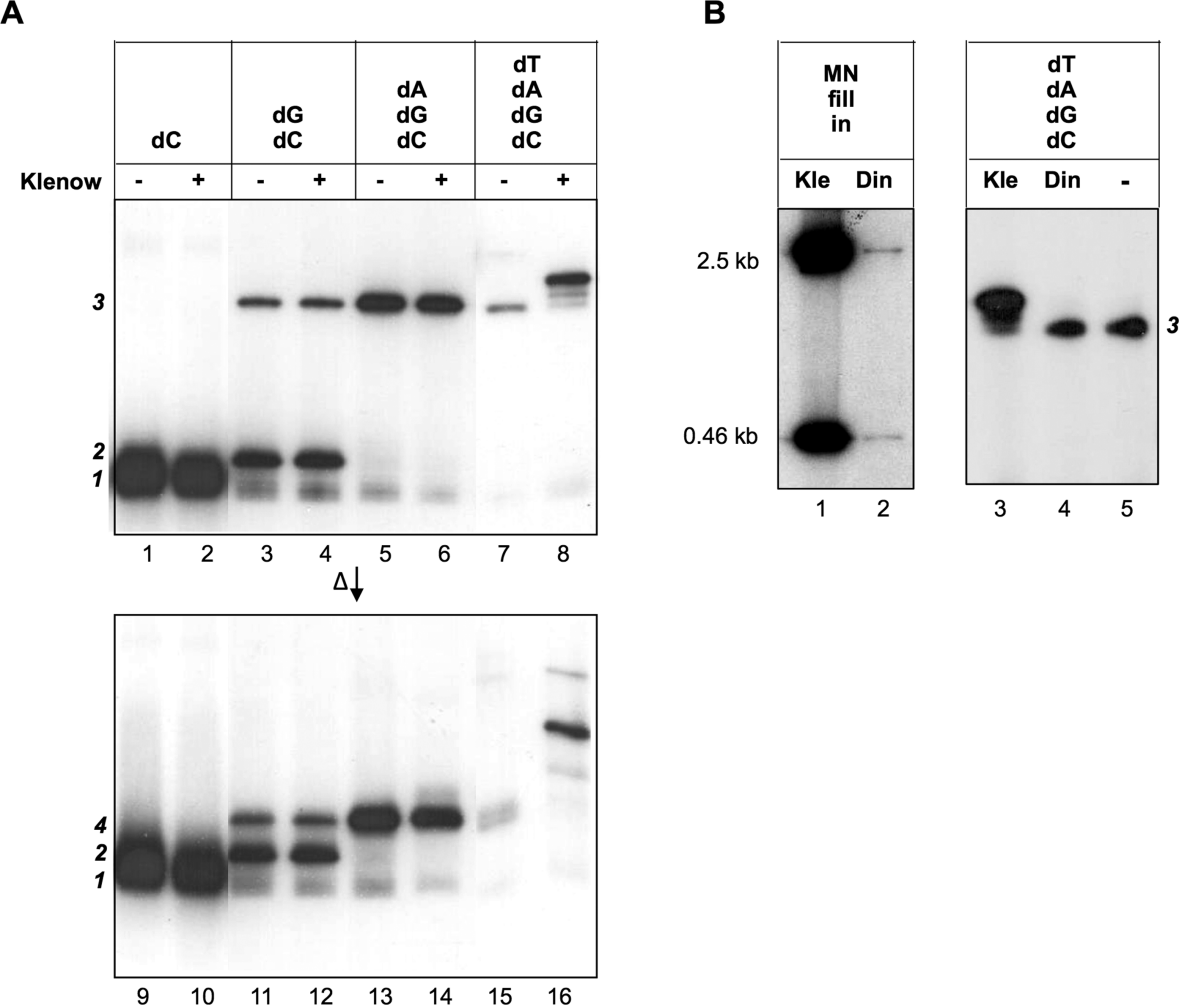
Effect of added DNA polymerases. (**A**) *The effect of added Klenow fragment*. The template was the 35-nt telomere DNA (TO35). The reaction conditions were identical to Figure 2B except for 4 h of reaction time and the addition of Klenow fragment (0.2 units; even-numbered lanes). The products were electrophoresed in 15% SDS-PAGE directly (upper panel) or after heat denaturation (lower panel). [α^32^P]-dCTP (0.165 μM) was supplemented with 10 μM non-radioactive dCTP, dATP, dGTP and dTTP as indicated. (**B**) *The effect of added DinB1. Left panel*. The polymerase activity of purified DinB1 was demonstrated by the fill in reaction on the MulI–NdeI restriction fragments (‘MN’; Figure 2B). The reaction conditions were identical to those in Figure 2B. *Right panel*. The extension activity on the *in vitro* deoxynucleotidylated product by DinB1 was tested under the same conditions as in (**A**). ‘Kle’, Klenow fragment; ‘Din’, DinB1; ‘–’, none added.

To test whether the13-nt primer might be further extended by DNA polymerase in our *in vitro* system, we added Klenow fragment of *E. coli* Pol I to the reactions (Figure 6A). No extension was detected, when the reactions contained only dCTP (lane 2), dCTP and dGTP (lane 4), or dCTP, dGTP and dATP (lane 6). However, when the reaction contained all four dNTPs (lane 8), addition of Klenow fragment significantly increased the lengths of the labeled products. The observed extension by the inclusion of dTTP was expected, because dTTP allowed the synthesis to pass the 14th nt (Figure 5C). The extension synthesis by Klenow fragment, however, was very inefficient, and was observed only after prolonged incubation (4 h in Figure 6). The purified DinB1 polymerase was also tested under the same conditions for extension synthesis. No extension was observed in repeated tests (Figure 6B).

### Telomere-association protein Tac also deoxynucleotidylates its cognate terminal protein Tpc

**Figure 7.**
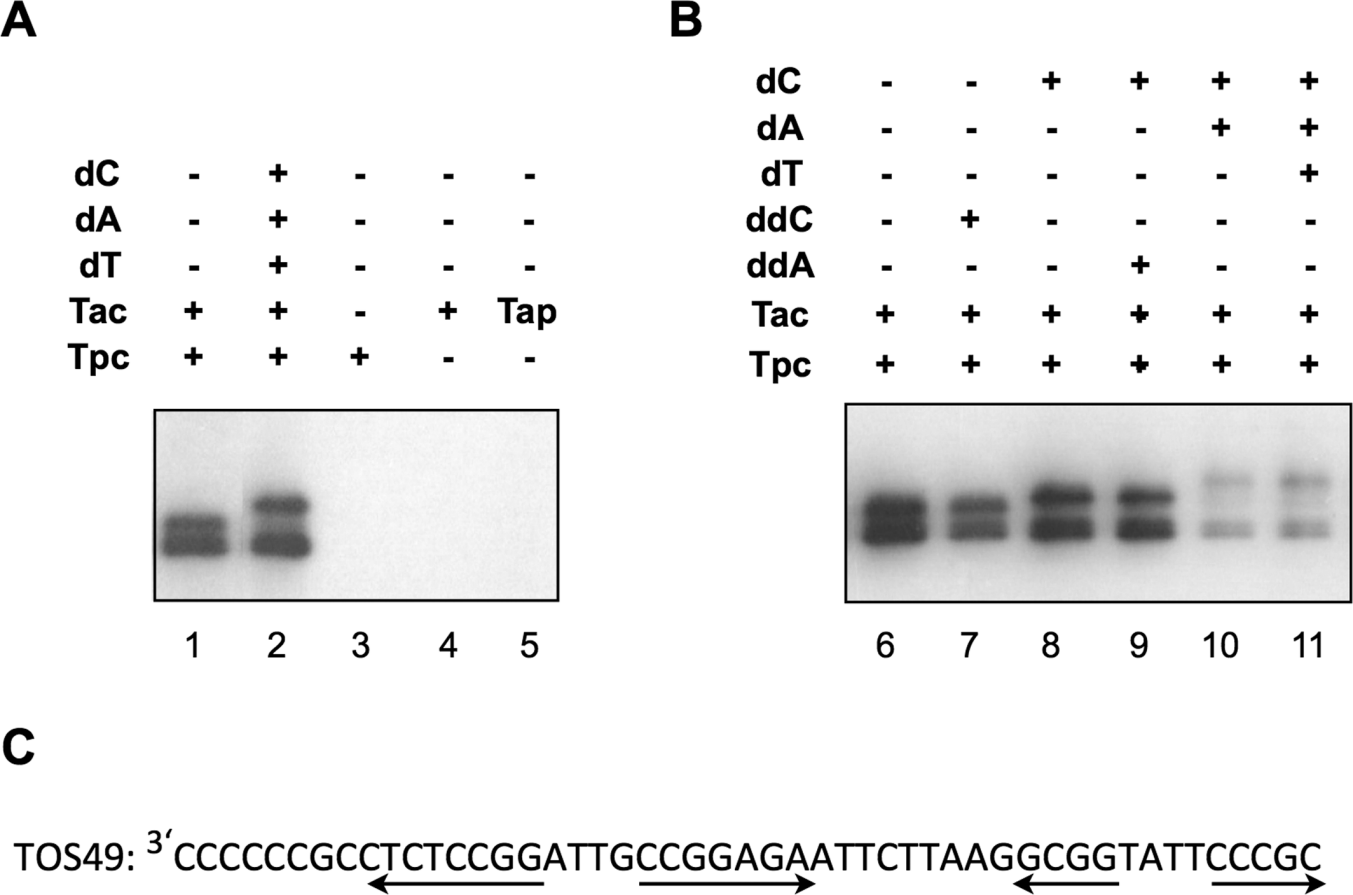
Deoxynucleotidylation of Tpc by Tac of SCP1 plasmid. (**A**) *The roles of Tac and Tpc*. In the reaction, Tap and Tpg were singly or doubly replaced by Tac and Tpc, respectively. (**B**) *The effect of ddCTP (‘ddC) and ddATP (‘ddA’)*. (**C**) *The 3′ single-strand overhang of SCP1 telomere*. The first 49 nt (‘TOS49’) was used as the template in (A) and (B). The palindromes are indicated by the divergent arrows.

SCP1 plasmid possesses atypical telomeres capped by atypical terminal proteins (Tpc) and telomere-association protein (Tac) (Figure 7C) (10). We tested whether Tac might also exert a similar deoxynucleotidylation function *in vitro*. In the *in vitro* deoxynucleotidylation system, Tap and Tpg were replaced by purified Tac and Tpc, the *S. lividans* telomere template by a 49-nt telomeric overhang (TOS49) of the SCP1 telomere, and radioactive dCTP by radioactive dGTP. The results (Figure 7A) showed that Tac was necessary and sufficient for deoxynucleotidylation of Tpc (lanes 1, 3, 4), and that it could not be replaced by Tap (lane 5).

The added dGTP was expected to extend up to six nucleotides (Figure 7C). Further extension was observed, when the other three dNTPs were also added (lane 2). The nucleotide following the G-string is a C (Figure 7C). Thus, as expected, elongation was observed, when dCTP (Figure 7B, lane 8), but not ddCTP (lane 7), was added to the reaction. Similarly, further elongation was observed when dATP (lane 10), but not ddATP (lane 9) was added to the reaction. Interestingly, the addition of the fourth nucleotide triphosphate, dTTP, did not cause detectable further elongation (lane 11). This suggested a steric limitation for Tac to extend the Tpc-primed oligonucleotide beyond a relatively short length, as in the case of deoxynucleotidylation of Tpg by Tap,

## DISCUSSION

Linear replicons face a biochemical complication, when internally initiated replication reaches the telomeres. The universal 5′ to 3′ directionality of the DNA synthesis prevents the duplication of the 3′ ends, and leave single-stranded overhangs, which, if not dealt, with would result in shortening of the telomeres with each round of replication (22). Eukaryotic cells use telomerases to extend the 3′ ends to counterbalance the telomere shortening (23). Linear viral and plasmids in eukaryotes or bacteria do not use this strategy. Instead they have evolved widely divergent mechanisms to fill up the 3′ single-strand gaps, such as homologous recombination in T4 (24) and T7 (22) phages, site-specific recombination in linear replicons with hairpined ends, and protein-primed DNA synthesis in *Streptomyces* and certain related actinomycetes (25).

In this study, we show that in our *in vitro* system, the telomere-associated proteins, Tap and Tac, were able to deoxynucleotidylate the TPs, Tpg and Tpc, respectively, and continue DNA synthesis to a short length. In the case of Tap, it extended the oligomer to 13 nt, stopping at the junction between Palindromes I and II. The exact stopping point of chain growth in the Tpc-primed synthesis by Tac was not determined.

These discoveries were surprising in two aspects: Firstly, the telomere-associated proteins use the TPs as the primers for DNA synthesis, but not the 5′ overhangs of the restriction fragments added to the reactions. In this fashion, they appear as primer-specific DNA polymerases. Secondly, these DNA polymerases only produce oligomers of limited lengths. The two classes of telomere-associated proteins of *Streptomyces* share no sequence homology between them or with any other proteins in the database. There is not any DNA polymerase motif in them. Yet, they possess the ability to deoxynucleotidylate the terminal proteins and synthesize a short oligomer. Thus, these proteins represent a new class of DNA polymerases.

Such two-step protein-primed DNA synthesis is also found in replication of viruses. In replication of adenoviruses and ϕ29, a single Family-B DNA polymerase catalyzes both the deoxynucleotidylation and the continuous synthesis. The ϕ29 TP has a high affinity for the DNA polymerase, but it dissociates from the polymerase after the an oligonucleotide of 6–10 nt is synthesized (26). The release of the TP from the polymerase triggers a transition from an initiation to elongation phase, in which a stable template–primer complex is formed, and synthesis proceeds with high processivity. This is supported by the co-crystal structure of ϕ29 TP–polymerase complex (27).

Similar two-step synthesis is also observed in replication of hepadnaviruses. The replication of Hepatitis B virus is initiated by viral P protein, which contains a reverse transcriptase (RT) domain and a TP domain. The latter acts as a primer for the initiation of synthesis at a bulged region of a RNA template. A short (3–4 nt) oligomer is synthesized that remained covalently linked to a Tyr of the TP domain (reviewed in 13). This oligonucleotide serves, after a template switch to the 3′ proximal direct repeat, to initiate full-length (−)-strand synthesis.

It is possible that the polymerase activities observed in our *in vitro* system require the binding of the telomere-associated proteins with the respective TPs. A reverse transcriptase motif (thumb domain) is present in the Tpg sequences, whereas a DNA-binding domain is found in Tap. Possibly, the polymerase activity only emerges when the Tpg–Tap or Tpc–Tac heterodimer is formed. In this model, these Tpg–Tap and Tpc–Tac heterodimers are analogous to the P-protein of hepadnaviruses, which possess a TP domain linked to a polymerase (reverse transcriptase) and an Rnase H domain. During replication of the hepadnaviruses, the polymerase domain also produces a short (3- to 4-nt) oligomer using the TP domain as the primer.

It is plausible to view P-protein of Hepatitis B structurally and functionally as a fusion of the TP and telomere-associated protein pair of *Streptomyces*—with its N-terminal TP domain corresponding to the TP of *Streptomyces* and its polymerase domain corresponding to the telomere-associated protein of *Streptomyces*. Moreover, both the polymerase domain and the Tap bind to a hairpin structure formed by the template polynucleotide chain. Tap binds strongly (*K*_a_ = 1 × 10^7^) and specifically Palindromes II and III of the single-stranded telomere DNA (9). It is possible that further extension of the oligonucleotide may require the removal of Tap from these sites. Alternatively, the tight binding between Tap and Tpg prevents Tap from moving away from Tpg for further oligomer extension.

The fact that these DNA polymerases use the TPs as primers for synthesis of a short oligonucleotides, which in term act as primers for further DNA synthesis creates a potential conceptual and nomenclature issue. Two primers are involved: the first (primary) primer being protein, and the second (secondary) DNA. No RNA is involved.

TP-primed nascent oligomer in *Streptomyces* must be extended to fill the 3′ single-strand overhangs to complete the replication. We postulate that the extension of the nascent oligomer is carried out by DinB1 or DinB2 but not by Pol I, because replication of linear chromosomes and linear plasmids in *Streptomyces* requires the former and not the latter (28). However, the addition of DinB1 to our *in vitro* system did not extend the oligomer, whereas the addition of Klenow fragment extended the oligomer but at a very low efficiency. It is probable that a helicase is required to remove the Tap bound at Palindrome II to clear the path of elongation, or perhaps a clamp and/or clamp loader is required to position DinB1 properly for the extension synthesis.

The discovery we made in this study was surprising, because it was totally unexpected. However, once it was made, it started to make sense when we compared it to the other TP-priming systems. Despite their divergent evolutional paths, they appear to share a similar biochemical mechanism in the unusual situation of DNA synthesis using a protein as the primer. Yet there is no sequence homology among (i) the telomere sequences, (ii) the TP sequences or (iii) the accessory proteins/DNA polymerases in different systems. It is amazing that such unorthodox systems have evolved independently and convergently in different kingdoms. These variations on the same theme are of great biochemical and evolutionary interest.

## SUPPLEMENTARY DATA

Supplementary Data are available at NAR Online.

SUPPLEMENTARY DATA
