# Supplementary material for: Telomere-associated proteins add deoxynucleotides to terminal proteins during replication of the telomeres of linear chromosomes and plasmids in Streptomyces
Source: Nucleic Acids Res. 2015 Apr 16;43(13):6373–83. doi: 10.1093/nar/gkv302 (PMC4513846; doi:10.1093/nar/gkv302)
Supplement: SUPPLEMENTARY DATA [file supp_43_13_6373__index.html]

Telomere-associated proteins add deoxynucleotides to terminal proteins during replication of the telomeres of linear chromosomes and plasmids in Streptomyces — SUPPLEMENTARY DATA 

# Telomere-associated proteins add deoxynucleotides to terminal proteins during replication of the telomeres of linear chromosomes and plasmids in *Streptomyces*

## SUPPLEMENTARY DATA

**Files in this Data Supplement:**

- SUPPLEMENTARY DATA
