# Supplementary material for: Telomere-associated proteins add deoxynucleotides to terminal proteins during replication of the telomeres of linear chromosomes and plasmids in Streptomyces
Source: Nucleic Acids Res. 2015 Apr 16;43(13):6373–83. doi: 10.1093/nar/gkv302 (PMC4513846; doi:10.1093/nar/gkv302)
Supplement: SUPPLEMENTARY DATA [file supp_gkv302_nar-00172-x-2015-File004.pdf]

Table S1 PCR Primers for production of telomere DNA

Product	Sequences	Note
Fig. 2A	ATAGAGGGCCCGCGGAGCGGGTACCC<br>AGCGCAGCGGTCACCTCG	*Hae*III site underlined
Fig. 2B	ACGCGTCGACCCGCGGAGCGGGTACCC<br>AGCGCAGCGGTCACCTCG	Overlapping *Mlu*I and *Sal*I sites underlined
